# α-smooth muscle actin-positive fibroblasts correlate with poor survival in hepatocellular carcinoma

**DOI:** 10.3892/ol.2013.1720

**Published:** 2013-11-29

**Authors:** JIGNESH G. PARIKH, ANAND KULKARNI, CAMEILA JOHNS

**Affiliations:** 1 Department of Pathology, University of Tennessee Health Science Center, Memphis, TN 38163, USA; 2 Department of Pathology, Veterans Affairs Medical Center, Memphis, TN 38104, USA

**Keywords:** activated fibroblasts, hepatocellular carcinoma, survival

## Abstract

The current case-control study was conducted to assess the value of activated fibroblasts with high α-smooth muscle actin (α-SMA) expression as an indicator of survival in hepatocellular carcinoma (HCC) patients. In total, 47 patients diagnosed with HCC who underwent a liver biopsy at the Veterans Affairs Medical Center (Memphis, TN, USA) between January 2000 and December 2009, and 10 age- and gender-matched controls with normal liver biopsy samples were included in the study. The immunohistochemical staining for α-SMA was performed and classified qualitatively and quantitatively within tumor stroma and perisinusoidal spaces. HCC patients with high qualitative and quantitative expression of α-SMA revealed a statistically significant negative correlation with 3-year [odds ratio (OR), 0.021 and 0.111; P=0.001 and 0.0302, respectively] and 1.5-year (OR, 0.040 and 0.051; P=0.0330 and 0.0492, respectively) survival rates in comparison with low expression. The results of the present study demonstrated a predictive role of high qualitative and quantitative expression in activated fibroblasts for poor survival in patients with HCC.

## Introduction

Activated fibroblasts or myofibroblasts with α-smooth muscle actin (α-SMA) expression are considered to be the main cellular constituents of reactive stroma in a number of solid tumors (1). These activated fibroblasts have been proposed to be important in promoting tumor progression and metastasis by releasing growth factors, extracellular matrix proteins and angiogenic factors (2,3). The hepatic stellate cell (HSC), an important hepatic stromal cell, is known to be activated or transdifferentiated into a myofibroblast-like cell through intercellular communication between HSCs and damaged hepatocytes in various liver tumors, including hepatocellular carcinoma (HCC) (4). These activated HSCs demonstrate a high expression of α-SMA and are considered to be a vital stromal component in HCC (5). Increasing evidence has indicated that activated HSCs play a critical role in promoting HCC cell proliferation and invasiveness (6,7). In addition, HSCs are now being considered as a new potential therapeutic target in patients with HCC (3). To the best of our knowledge, no direct evidence has been identified documenting a correlation between fibroblast activation and survival in HCC patients. The aim of the present study was to assess the value of activated fibroblasts with high α-SMA expression as an indicator for survival in patients with HCC.

## Patients and methods

**Figure 1:**
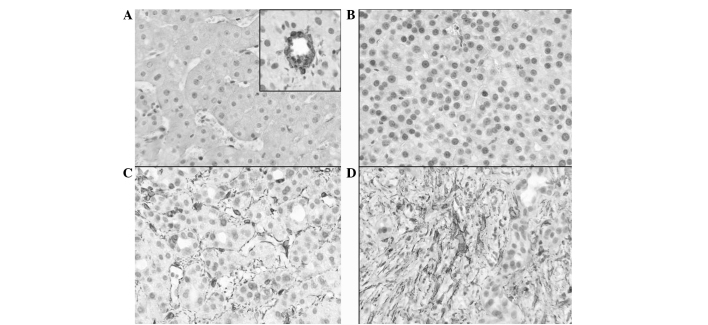
Expression of α-SMA in HCC. (A) Control tissues revealing positive staining for α-SMA mainly in the perisinusoidal spaces and VSMC (inset). (B) Grade +1 staining (qualitatively and quantitatively); staining intensity was considerably lower than in the VSMC, with <10 positive cells/hpf. (C and D) Grade +3 staining (qualitatively and quantitatively); staining intensity was similar to that of VSMC, with >20 positive cells/high power field (magnification, ×400). α-SMA, α-smooth muscle actin; HCC, hepatocellular carcinoma; hpf, high-power field; VSMC, vascular smooth muscle cells.

A case-control study was performed at the Veterans Affairs Medical Center (Memphis, TN, USA) following appropriate Institutional Review Board approval. A review of the computerized hospital records was conducted to identify the cases of patients diagnosed with HCC who had undergone a liver biopsy between January 2000 and December 2009. A total of 10 age- and gender-matched cases with no histopathological abnormality on liver biopsy were also identified and used as controls. The clinical information with regard to age and survival period were obtained for all study cases. The immunohistochemical staining for α-SMA was performed on the formalin-fixed, paraffin-embedded tissue sections of all study and control cases. The α-SMA staining in the HSCs within the tumor stroma and perisinusoidal spaces was qualitatively classified into the following 4 groups compared with vascular smooth muscle cells (VSMCs): 0, no staining; +1, staining intensity considerably lower than VSMCs (Fig. 1B); +2, staining intensity lower than VSMCs; and +3, staining intensity similar to that of VSMCs (Fig. 1C and D). In addition, the α-SMA staining was quantitatively classified into the following 4 groups: 0, ≤1 positive cell/high-power field (hpf); +1, <10 positive cells/hpf (Fig. 1B); +2, 10–20 positive cells/hpf; and +3*,* >20 positive cells/hpf (Fig. 1C and 1D). Qualitative and quantitative classification was performed by a fellowship-trained gastrointestinal pathologist without matching knowledge of the clinical data. For statistical analysis, grades 0 and +1 were categorized as low expression and grades +2 and +3 as high expression.

## Results

A total of 47 patients (age range, 45–85 years; mean age, 64.45 years) were diagnosed with HCC between January 2000 and December 2009. All the study subjects subsequently succumbed to their condition. The survival period following diagnosis ranged between 1 and 94 months (mean survival, 19 months). All the study and control subjects were male. Positive staining for α-SMA in control tissue was mainly observed in VSMCs (Fig 1A; inset) and in extremely few cells in the perisinusoidal spaces (Fig. 1A). High qualitative (strong) and quantitative (extensive) expression of α-SMA was revealed in 41 and 42 of the 47 patients, respectively. Low qualitative (weak) and quantitative (sparse) expression of α-SMA was revealed in 6 and 5 of the 47 patients, respectively. Strong and extensive expression of α-SMA revealed a statistically significant negative correlation with the 3-year [OR, 0.021 and 0.111; 95% confidence interval (CI), 0.002–0.234 and 0.015–0.810; P=0.001 and 0.0302, respectively] and 1.5-year (OR, 0.040 and 0.051; 95% CI, 0.002–0.771 and 0.002–0.990; P=0.0330 and 0.0492, respectively) survival rates in comparison with weak and sparse expression (Table I). No significant correlation was identified between age and α-SMA expression.

## Discussion

The interaction of tumor cells with surrounding stromal cells has been recognized to promote tumor development by affecting cell proliferation, survival and invasiveness (7). As important hepatic stromal cells, HSCs have been considered to play a key role in liver fibrosis when excessively activated in various liver diseases, including HCC (8). HCC is one of the most common types of malignant cancer in humans, with an overall 5-year survival rate of only 3% in the United States (9). Surgical resection is the choice of treatment for HCC, however, this is not feasible when patients present in late stages of the disease (10). In addition, radiotherapy and chemotherapy are not effective in advanced disease (10). Activated HSCs that express extremely high levels of α-SMA have emerged as potent suppressors of hepatic immunity by affecting T-cell responses and thus, play a vital role in the progression of HCC (6,7,11,12). Previous studies have also indicated that activated HSCs are potential targets for HCC treatment (4).

The current study revealed that the detection of α-SMA-expressing fibroblasts in HCC provides valuable prognostic information. As α-SMA is a routinely used and relatively inexpensive immunohistochemical stain, the majority of pathology laboratories efficiently employ α-SMA to predict future survival in HCC patients. The present study also supports the role of α-SMA expressing activated HSCs in promoting carcinogenesis, which is considered to be a possible target for future antitumor therapy in HCC.

In the present study, due to lack of follow-up staging information for all the patients, the results were not corrected for stage, metastasis and other factors. Due to this same reason, it was not possible to determine whether α-SMA is predictive of metastasis-related survival or is an independent predictive factor. Multicenter-based large studies with detailed and frequent staging evaluation data, which enables multifactorial analysis, are recommended to improve the present understanding of the role of α-SMA-expressing fibroblasts for predicting poor survival in HCC.

In conclusion, the current study demonstrated a predictive role of strong and extensive α-SMA expression in activated fibroblasts for poor survival in HCC patients. Strong expression of α-SMA is an excellent marker to anticipate poor 3-year survival (OR, 0.021; 95% CI, 0.002–0.234; P=0.001). Qualitative α-SMA expression levels in activated fibroblasts appear to present an improved marker for predicting survival compared with quantitative expression.

## Figures and Tables

**Table I:** Qualitative and quantitative expression of α-SMA in HCC.

		α-SMA staining intensity				α-SMA-positive HSC			
							
Characteristics	n, (%)	High, n (%)	Low, n (%)	OR (95% CI)	P-value	High, n (%)	Low, n (%)	OR (95% CI)	P-value
Age, years 1									
≤62	23 (49)	18 (38)	5 (11)	0.156	0.103	19 (40)	4 (9)	0.206	0.174
>62	24 (51)	23 (49)	1 (2)			23 (49)	1 (2)		
3-year survival									
>3	9 (19)	4 (8)	5 (11)	0.021 (0.002–0.234)	0.0016	6 (13)	3 (6)	0.111 (0.015–0.810)	0.0302
≤3	38 (81)	37 (79)	1 (2)			36 (77)	2 (4)		
1.5-year survival									
>1.5	20 (43)	14 (30)	6 (13)	0.040 (0.002–0.771)	0.0330	15 (32)	5 (11)	0.051 (0.002–0.990)	0.0492
≤1.5	27 (57)	27 (57)	0 (0)			27 (57)	0 (0)		

α-SMA, α-smooth muscle actin; HCC, hepatocellular carcinoma; OR, odds ratio; CI, confidence interval.
